# Ginsenoside Rg1 controls CKLF1-mediated apoptosis to reduce hypoxic/reoxygenation injury in HT22 cells

**DOI:** 10.3389/fphar.2025.1525605

**Published:** 2025-04-29

**Authors:** Jinping Liang, Xuan Zhu, Feng Li, Yan Yang, Yuchen Zhu, Shasha Liu, Yang Sun, Boyu Kuang, Junpeng Long, Qian Yan, Yuting Lin, Qidi Ai, Yantao Yang

**Affiliations:** ^1^ Hunan Engineering Technology Center of Standardization and Function of Chinese Herbal Decoction Pieces, College of Pharmacy, Hunan University of Chinese Medicine, Changsha, China; ^2^ Department of Pharmacy, Changsha Hospital for Maternal and Child Health Care Affiliated to Hunan Normal University, Changsha, China

**Keywords:** ginsenoside Rg1, OGD/R, CKLF1, apoptosis, inflammatory response

## Abstract

**Background:**

Stroke is a prevalent and debilitating neurodegenerative condition. Ginsenoside Rg1 has demonstrated neuroprotective properties in the context of stroke. The upregulation of chemokine-like factor 1 (CKLF1) observed in ischemic stroke positions CKLF1 as a promising therapeutic target. However, limited research has explored whether Rg1 can mitigate oxygen-glucose deprivation/reoxygenation (OGD/R)-induced apoptosis in HT22 cells through the modulation of CKLF1.

**Methods:**

In this study, Na_2_S_2_O_4_ was used to treat HT22 cells to establish the OGD/R model. The effects of different concentrations of Rg1 on cell viability were firstly determined by CCK-8 assay to determine its safe administration range. Subsequently, the level of oxidative stress was assessed by detecting LDH release and antioxidant indexes (CAT, SOD, MDA). Western blotting was used to analyse the expression of CKLF1 and apoptosis-related proteins, and TUNEL staining was used to quantify the apoptosis rate. To explore the cell-cell interactions, a Transwell co-culture system of HT22 and BV-2 cells was established.

**Results:**

In this study, the optimal parameters for the OGD/R model were determined: 25 mmol/L Na_2_S_2_O_4_ treatment for 2.5 h followed by 2.5 h of reoxygenation, and a cell inoculation density of 1 × 10^5^ cells/mL for 1 day of culture. Based on the safety assessment, 5, 25, and 50 μmol/L Rg1 were selected for intervention. Rg1 significantly decreased LDH release (*P* ≤ 0.05) and MDA content (*P* ≤ 0.05) and alleviated oxidative stress. Western blotting showed that Rg1 dose-dependently downregulated the expression of CKLF1 (*P* ≤ 0.05) and inhibited Caspase-3 and other apoptotic protein activation. In the HT22/BV-2 co-culture system, Rg1 inhibited microglia activation, as shown by reduced NO and IL-1β secretion (*P* ≤ 0.05).

**Conclusion:**

Rg1 attenuates OGD/R injury, reduces oxidative stress and apoptosis in HT22 cells by inhibiting CKLF1 expression and alleviates the inflammatory response in activated BV-2 cells, showing therapeutic potential.

## 1 Introduction

Stroke is a major global health challenge due to its acute onset and its significant rates of morbidity, disability, and mortality (Tu et al., 2023). Both non-modifiable factors—such as age, gender, race, and genetic predisposition—and modifiable factors—including antiphospholipid syndrome, hypertension, diabetes, hyperlipidemia, and unhealthy lifestyle choices—contribute to its development (Barthels and Das, 2020). The pathophysiology of ischemic stroke is complex, involving cellular excitotoxicity, mitochondrial dysfunction, ionic imbalances, acid-base disturbances, neuroinflammation, blood-brain barrier (BBB) disruption, and dysregulated long noncoding RNA (lncRNA) expression (Akella et al., 2019; Datta et al., 2020; Qin et al., 2022). Despite advances in understanding these mechanisms, current treatment options for cerebral ischemia remain limited and challenging to implement effectively, given the progressive nature of the condition. Intravenous thrombolytic agents, such as recombinant tissue-type plasminogen activators, are the primary therapeutic option. Additionally, recent studies have explored arterial thrombolysis, encompassing both chemical thrombolysis and mechanical thrombectomy, as potential alternative interventions (Zhao et al., 2022).

CKLF1 is widely expressed in humans and plays key roles in various biological processes that may contribute to the physiological process of human growth. The expression levels of CKLF1 differ in adults and fetuses, and its aberrant expression may be a predictor of pathological states. Its diverse chemotactic effects make it a promising target for stroke therapy (Li Y. et al., 2023). In experimental rat models of focal cerebral ischemia, the CKLF1 antagonist C19 peptide has demonstrated efficacy in reversing CKLF1-mediated cerebral ischemia, as evidenced by reductions in infarct size and cerebral water content (Kong et al., 2012). It was found that CKLF1 participates in neuronal apoptotic process by regulating apoptotic signalling pathway in cerebral ischemia-reperfusion injury. Anti-CKLF1 antibody significantly inhibited the phosphorylation of Akt protein, which in turn downregulated the expression of the pro-apoptotic factor Bax, and at the same time upregulated the expression of the anti-apoptotic protein Bcl-2, which led to a significant increase in the Bcl-2/Bax ratio. This cascade of regulatory effects ultimately inhibited the activation of caspase-3, a key effector of apoptosis, and thus showed neuroprotective effects against cerebral ischaemia (Kong et al., 2014b). Treatment with CKLF1 antagonists has been shown to suppress the production of inflammatory mediators, including TNF-α, IL-1β, MIP-2, and IL-8, as well as the expression of adhesion molecules such as ICAM-1 and VCAM-1. This anti-inflammatory effect is achieved by inhibiting the MAPK pathway, which subsequently reduces neutrophil infiltration (Kong et al., 2014a). Additionally, CKLF1 expression is significantly upregulated during the early stages of ischemic stroke and contributes to the polarization of microglia/macrophages towards the pro-inflammatory M1 phenotype, exacerbating the release of pro-inflammatory factors and intensifying the inflammatory response (Chen et al., 2019).

Ginsenoside Rg1, a key bioactive compound derived from Panax ginseng, has shown significant neuroprotective properties by maintaining the structural and functional integrity of cells through its antioxidative, anti-apoptotic, and anti-inflammatory actions (W et al., 2018). Studies have shown that Rg1 exerts its anti-apoptotic effects through multiple pathways, it can effectively inhibit the release of cytochrome c from the mitochondrial membrane gap, regulate the NF-κB/Bcl-2 signalling pathway by down-regulating the expression of Bax protein and inhibiting the activation of caspase-9, furthermore, Rg1 can activate the PI3K/Akt signalling pathway and induce the phosphorylation of pro-apoptotic protein Bad, which in turn inhibits its pro-apoptotic activity (Li et al., 2018; Huang et al., 2023). Recent studies have explored the potential of Rg1 as an adjunctive therapy for stroke, particularly in combination with treatments such as Xuesaitong injection (Zhou et al., 2021) or Xueshuantong (Wang et al., 2022). Rg1 demonstrates a multifaceted therapeutic effect, both by preventing the onset of stroke and promoting recovery of the nervous system. Given the observed alterations in CKLF1 expression during stroke, our study aimed to investigate whether Rg1 could act as an inhibitor of CKLF1, thereby suppressing its expression and exerting a neuroprotective effect on neural cells. Furthermore, we explored whether Rg1 could mitigate apoptosis induced by stroke through the modulation of CKLF1.

This study aims to employ HT22 neuronal cells as an *in vitro* model to simulate cellular conditions representative of stroke. To evaluate the neuroprotective effects, nimodipine will be used as a positive pharmacological control, while varying concentrations of Rg1 will be administered as interventions. Different concentrations of Rg1 will be systematically tested to investigate its potential therapeutic benefits in the context of stroke.

## 2 Materials and methods

### 2.1 Antibodies and reagents

The Rg1 compound (molecular formula: C_42_H_72_O_14_; molecular weight: 801.01; purity ≥98%) was procured from Shanghai Ronghe Pharmaceutical Technology Development Co., Ltd. (Shanghai, China). Na_2_S_2_O_4_ was procured from China National Pharmaceutical Group Co., Ltd. (Beijing, China). Nimodipine was procured from Yabao Pharmaceutical Group Co., Ltd. (Shanxi China). Antibodies against CKLF1, Caspase-3, Bax, Bcl-2, Cleaved PARP1, Cytochrome C (Cyt-c) antibodies were purchased from Abcam (Cambridge, MA, United States).

### 2.2 OGD/R injury model

#### 2.2.1 Time of hypoxia/reoxygenation

HT22 cells were cultured in sugar-free serum-free DMEM (Procell Life Science & Technology Co., Ltd., Wuhan, China) and subjected to different concentrations of Na_2_S_2_O_4_ including 0, 10, 20, 40, 60, 80, 100, 150, 200, and 400 mmol/L. The incubation periods were set as follows: 1.5, 2, 2.5, 3, and 3.5 h in a cell incubator with 5% CO2 at 37°C. Subsequently, the cells were incubated with high-sugar DMEM (Procell Life Science & Technology Co., Ltd., Wuhan, China) for 3.5, 3, 2.5, 2, and 1.5 h. Cell viability was determined using the MTT (BioFroxx, Germany) assay.

#### 2.2.2 Concentration of hypoxia agent

HT22 cells were cultured in a cell incubator with 5% CO_2_ at 37°C and exposed to different concentrations of Na_2_S_2_O_4_, including 0, 5, 10, 15, 20, 25, and 30 mmol/L, for a duration of 2 h. Subsequently, the cells were incubated with high-sugar DMEM for 3 h. Cell viability was assessed using the MTT method.

#### 2.2.3 Density of cell culture

HT22 cells were seeded into 96-well plates at densities of 4 × 10^5^ cells/mL, 2 × 10^5^ cells/mL, 1 × 10^5^ cells/mL, 0.8 × 10^5^ cells/mL, 0.4 × 10^5^ cells/mL, and 0.1 × 10^5^ cells/mL per well. The cells were then cultured in a cell incubator with 5% CO_2_ at 37°C for 48 h. To induce OGD/R, the cells were treated with 20 mmol/L Na_2_S_2_O_4_ for 2 h, followed by replacement with high-sugar DMEM for 2.5 h. Cell viability was assessed using the MTT method.

#### 2.2.4 Time of cell culture

HT22 cells were cultured in a CO_2_ incubator at 37°C with 5% CO_2_ for 1, 2, and 3 days, respectively. To induce OGD/R, the cells were treated with 15 mmol/L Na_2_S_2_O_4_ for 2 h, followed by replacement with high-glucose DMEM for 3 h. Cell viability was assessed using the MTT method.

#### 2.2.5 Evaluation of screening methods and model validation

To assess the suitability of the model for high-throughput screening, the Z-factor was employed as a measure. Sixty wells were randomly chosen from a 96-well plate, with thirty wells serving as the negative control (normal modeling) and the remaining thirty wells as the positive control (normal modeling with the addition of a positive drug). The cell viability was assessed in triplicate using the MTT method, and the Z-values were calculated using Equation 1.

$$Z=1-\frac{{3\sigma }^{+}+{3\sigma }^{-}}{\left|\mu^{+}-\mu^{-}\right|}$$ (1)

Equation 1 μ^+^ is the mean of the positive control group, μ^−^ is the mean of the negative control group, σ^+^ is the standard deviation of the positive control, and σ^−^ is the standard deviation of the negative control group.

### 2.3 Screening of safe and effective concentrations of ginsenoside Rg1

#### 2.3.1 Ginsenoside Rg1 safe concentration screening

The control group consisted of HT22 cells that were cultured under routine conditions without any additional treatment. In the Rg1 administration group, HT22 cells were treated with various concentrations of Rg1 (0, 5, 10, 15, 25, 30, 50, 100, 200, and 400 μmol/L) and incubated for 24 h. The cell viability was assessed using the MTT method to determine the safe concentration range of the drug. This approach allowed for the evaluation of the effects of different doses of Rg1 on HT22 cell activity, providing valuable information regarding the suitable and tolerable concentrations for subsequent experiments.

#### 2.3.2 Ginsenoside Rg1 effective concentration screening

The control group consisted of HT22 cells that were cultured under routine conditions without any additional treatment. In the OGD/R group, HT22 cells were subjected to the established OGD/R model conditions, which were determined through previous screening. The OGD/R model involved subjecting the cells to a specific protocol to mimic the conditions of oxygen-glucose deprivation and subsequent reoxygenation. In the Rg1 administration group, HT22 cells that underwent OGD/R modeling were treated with various concentrations of Rg1 (0, 5, 10, 15, 25, 30, and 50 μmol/L) for 24 h. The cell viability was assessed using the MTT method to determine the effective concentration range of the drug. This experimental design allowed for the investigation of the potential protective effects of Rg1 on HT22 cells after OGD/R injury and the identification of suitable concentrations for subsequent experiments.

### 2.4 Cell culture

In order to simulate an *in vitro* cellular model of stroke, this study will be conducted by modeling commonly used HT22 cells, with nimodipine as a positive pharmacological control and low, medium, and high doses of Rg1 administered for testing. The experiments were carried out as follows: blank group, operated by OGD/R, but replacing Na_2_S_2_O_4_ with sugar-free serum-free DMEM; OGD/R group; positive drug group, treated according to the best OGD/R model conditions and added Nimodipine to give a final concentration of 25 μmol/L; low-dose-Rg1 group, 5 μmol/L Rg1+OGD/R; medium-dose-Rg1 group, 25 μmol/L Rg1+OGD/R; high-dose-Rg1 group, 50 μmol/L Rg1+OGD/R. Each group was incubated for 24 h in a cell incubator with 5% CO_2_ at 37°C.

### 2.5 Lactate dehydrogenase (LDH) activity testing

Levels of nerve cell damage were assessed using a LDH kit (Nanjing Jiancheng Bioengineering Institute, China), according to the manufacturer’s instructions. The optical density was measured using a microplate reader set to 450 nm.

### 2.6 Catalase from bovine liver (CAT) activity testing

Catalase activity was measured using a CAT kit (Nanjing Jiancheng Bioengineering Institute, China), in accordance with the manufacturer’s instructions. The optical density was determined using a microplate reader set to 405 nm.

### 2.7 Superoxide dismutase (SOD) activity testing

Cellular SOD activity was measured using a SOD kit (Nanjing Jiancheng Bioengineering Institute, China), following the manufacturer’s instructions. The optical density of the samples was determined using a microplate reader set to 450 nm.

### 2.8 Malonic dialdehyde (MDA) activity testing

Cellular levels of MDA were quantified using a MDA kit (Nanjing Jiancheng Bioengineering Institute, China) according to the manufacturer’s instructions. The optical density of the reaction mixture was measured at a wavelength of 532 nm using a microplate reader.

### 2.9 Western blot

Cellular lysates were prepared by treating cells with RIPA (NCM, China) buffer supplemented with protease inhibitors, followed by extraction of total cellular protein using high-speed centrifugation. The protein concentrations in the lysates were determined using a BCA kit (Nanjing Jiancheng Bioengineering Institute, China). Subsequently, the protein samples were subjected to SDS-PAGE using 10% polyacrylamide gels and transferred onto PVDF membranes (Millipore, USA) using a wet electro-transfer method. To prevent non-specific binding, the membranes were blocked with 5% skimmed milk powder and then incubated overnight at 4°C with primary antibodies (1/1000 of CKLF1, Bcl-2, Bax, Cleaved PARP1 and 1/5000 of Cyt-c) specific to the target proteins. The next day the blot was washed with TBST for 3 × 5 min and the corresponding secondary antibody was added. The blot was then incubated for 1 h at room temperature. The membrane was washed with TBST and fully covered with ECL chemiluminescence kit (NCM, China) and finally placed into the amersham imager 600 chemiluminescence imager.

### 2.10 Immunofluorescence (IF)

IF staining was performed to assess the expression of CKLF1 positive cells in HT-22 cells. HT22 were inoculated in 24-well plates with a disc on the bottom, treated with OGD/R and Rg1, fixed with 4% paraformaldehyde for 20 min at room temperature, and washed 3 times with PBS. The cells were then incubated with 0.3% TritonX-100 for 1 min at room temperature and washed 3 times with PBS before being incubated with primary antibody CKLF1 (1:100) at 4°C overnight. The next day, cells were allowed to stand for 30 min at room temperature before being washed 3 times in PBS and incubated with the corresponding secondary antibody for 1.5 h at room temperature. After being washed 3 times in PBS, the plates were blocked with an anti-fluorescence quenching blocker containing DAPI (Sigma-aldrich, China). Imaging was performed by confocal inverted microscopy (FV1000, Olympus, Tokyo, Japan) at ×40 magnification.

### 2.11 TUNEL staining

Apoptosis of HT22 cells was assessed by TUNEL assay using a one-step TUNEL apoptosis detection kit (Beyotime, China). HT22 was inoculated on a 24-well plate with a disc on the bottom, treated with OGD/R and Rg1, and then fixed with 4% paraformaldehyde for 20 min at room temperature and washed 3 times with PBS. The plates were then incubated with proteinase k for 1 min at room temperature, washed 3 times with PBS and then incubated with TUNEL assay solution for 2 h at 37°C. The plates were then incubated with Proteinase K for 1 min at room temperature and washed 3 times with PBS. At the end of the incubation, the film was washed 3 times with PBS and sealed with an anti-fluorescence quenching sealer containing DAPI. Imaging was performed by confocal inverted microscopy at ×20 magnification. All cell nuclei were stained with DAPI and apoptotic cell nuclei were stained with TUNEL. Apoptosis index = number of TUNEL-positive cells/total number of cells.

### 2.12 The co-culture model of neurons-microglia

A sterile transwell chamber (Corning, USA) was placed upside down in a sterile container, and HT22 cells were seeded on the outer surface of the transwell membrane. The assembly was incubated under tension for 5 h to allow HT22 cells to adhere to the membrane. Once the HT22 cells were fully attached, the setup was flipped over, and the culture was continued. Inside the transwell chamber, 1 mL of BV-2 cells suspended in conditioned DMEM medium was added to ensure parallel positioning of the inner and outer surfaces of the transwell. For the OGD/R group, the transwell chamber was separated, and HT22 cells were subjected to the optimal conditions for OGD/R modeling, while BV-2 cells were cultured under normal conditions. Following this, HT22 cells were treated with or without nimodipine and Rg1 at different concentrations (5, 25, 50 μmol/L), and the cultures were incubated using the transwell chambers for 24 h. This experimental design aimed to investigate the effects of nimodipine and Rg1 on HT22 cells in the presence of BV-2 cells using a transwell co-culture system.

### 2.13 Nitric oxide (NO) assay

Intracellular NO levels were determined as an indicator of nitrite content within the cells using the Griess method. In this assay, 50 μL of the sample was mixed with Griess reagent (Nanjing Jiancheng Bioengineering Institute, China) in each well, followed by incubation for 10 min at room temperature. The absorbance was then measured at 540 nm using a microplate reader.

### 2.14 Enzyme-linked immunosorbent assay (ELISA)

The levels of tumor necrosis factor alpha (TNF-α) and interleukin-1 beta (IL-1β) in each experimental group were quantified using ELISA kits (Multisciences, China) following the manufacturer’s instructions.

### 2.15 Statistics analysis

Statistical analysis of the data was conducted using GraphPad Prism 9.5.0 software. For comparisons between two groups of data, an independent samples t-test was employed. When comparing multiple groups of data, the one-way analysis of variance (ANOVA) test was utilized. The results are presented as the mean ± standard deviation. A value of *P* < 0.05 was considered statistically difference.

## 3 Results

### 3.1 OGD/R injury model

#### 3.1.1 Time of hypoxia/reoxygenation

**FIGURE 1:**
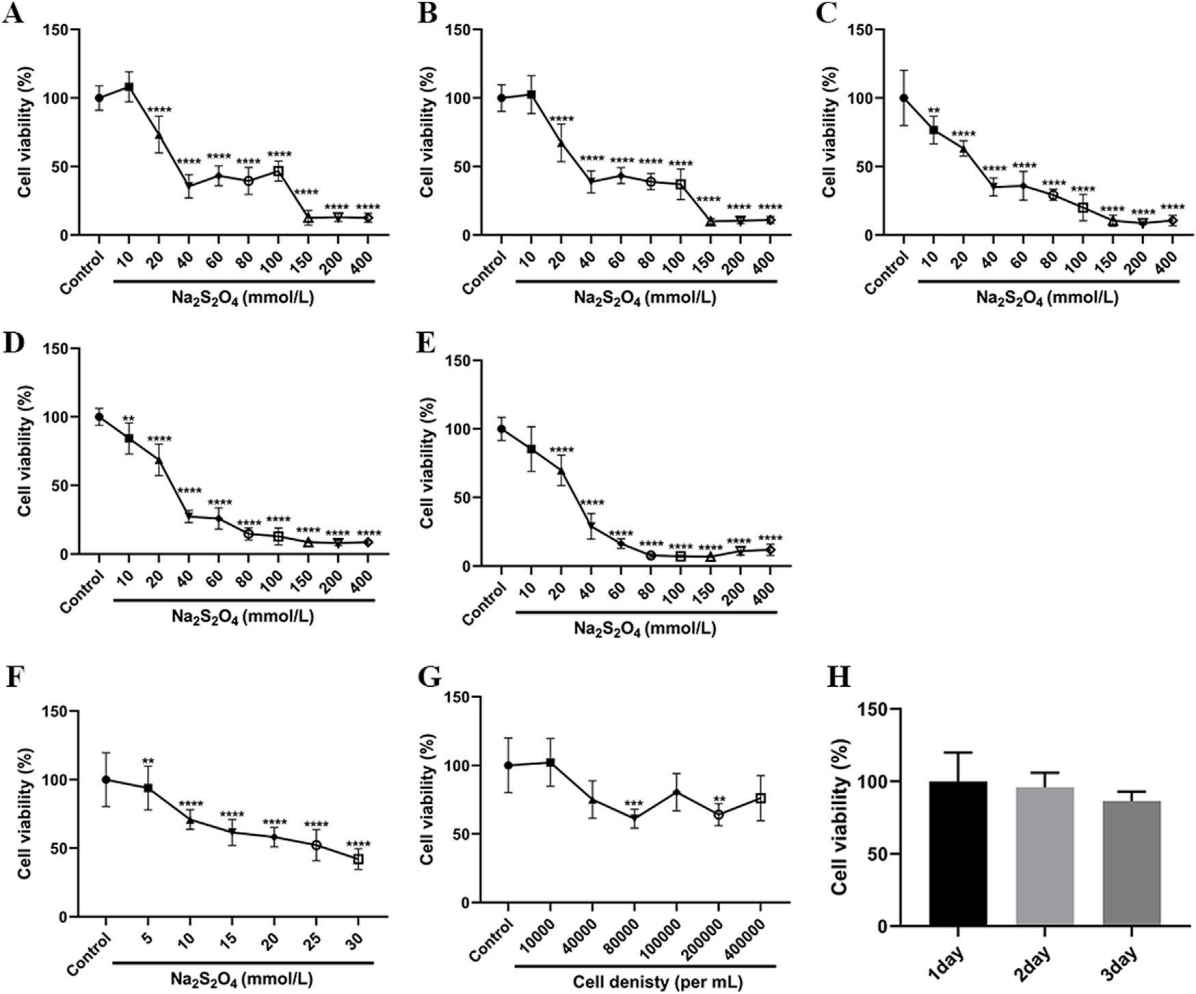
**(A–E)** Effect of different hypoxic reoxygenation time ratios on cell survival (A: 1.5 h/3.5 h; **(B)** 2 h/3 h; **(C)** 2.5 h/2.5 h; **(D)** 3 h/1.5 h; **(E)** 3.5 h/1.5 h) (n = 6). **(F)** Effect of different hypoxic agent concentration ratios on cell viability (n = 6). **(G)** Effect of different cell seeding plate densities on cell viability (n = 6). **(H)** Effect of different culture times on cell viability (n = 6). ** compared with control group, *P* ≤ 0.01; *** compared with control group, *P* ≤ 0.001; **** compared with control group, *P* ≤ 0.0001.

After subjecting HT22 cells to different ratios of OGD/R durations, cell viability was assessed using the MTT assay to determine the optimal duration for OGD/R. The results, shown in Figures 1A–E, indicated a linear relationship between cell viability and optical density (OD) values when the hypoxic agent concentration ranged from 0 to 40 mmol/L. Based on this correlation, the appropriate OGD/R duration was further evaluated. Specifically, at a hypoxic agent concentration of 25 mmol/L, cell viability was compared to that of the control group under various OGD/R time combinations. It was observed that at an OGD/R duration of 1.5 h of hypoxia followed by 3.5 h of reoxygenation, the decrease in cell viability was minimal, with a slight increase noted. However, at an OGD/R duration of 2.5 h/2.5 h, cell viability decreased maximally, reaching approximately 50% of the control group. Substantial reductions in cell viability were also observed at OGD/R durations of 3 h/2 h and 3.5 h/1.5 h. Considering both experimental outcomes and practical factors, an OGD/R duration of 2.5 h of hypoxia followed by 2.5 h of reoxygenation was selected for the subsequent experiments.

#### 3.1.2 Concentration of hypoxia agent

HT22 cells were exposed to different concentrations of hypoxic agents, and cell viability was assessed using the MTT assay to determine the optimal concentration for inducing hypoxia. Based on the results from the single-factor hypoxic reoxygenation experiment, significant changes in cell morphology and a marked decrease in cell viability were observed in the model group when the hypoxic agent concentration reached 40 mmol/L, indicating substantial cellular damage. However, concentrations above 60 mmol/L led to excessive cell damage, resulting in a further significant reduction in cell viability (Figure 1F). Consequently, a hypoxic agent concentration range of 0–30 mmol/L was selected for further studies. Among these, 25 mmol/L, which resulted in an OD50 value of HT22 cells comparable to the control group, was chosen as the optimal concentration for inducing hypoxic damage in subsequent experiments.

#### 3.1.3 Density of cell culture

HT22 cells were subjected to different seeding plate densities, and the cell viability was assessed using the MTT method to determine the optimal cell density (Figure 1G).

#### 3.1.4 Time of cell culture

HT22 cells were cultured for varying durations, and the cell viability was assessed using the MTT method to determine the optimal incubation time (Figure 1H).

#### 3.1.5 Model validation

Based on the results of the preliminary single-factor experiments, optimal conditions were selected for the subsequent validation experiments to assess cell viability. These conditions included an OGD/R time of 2.5 h of hypoxia followed by 2.5 h of reoxygenation, a hypoxic agent concentration of 25 mmol/L, a cell seeding density of 1 × 10^5^ cells/mL, and a culture duration of 1 day. Under these conditions, the cell survival rates were found to be 58.7%, 62.1%, and 63.5%, respectively. Additionally, the calculated Z-values were 0.54, which exceeds the threshold of 0.5, indicating that the model was both reasonable and consistent with the principles of high-throughput screening.

### 3.2 Ginsenoside Rg1 safe concentration range and effective concentration range

**FIGURE 2:**
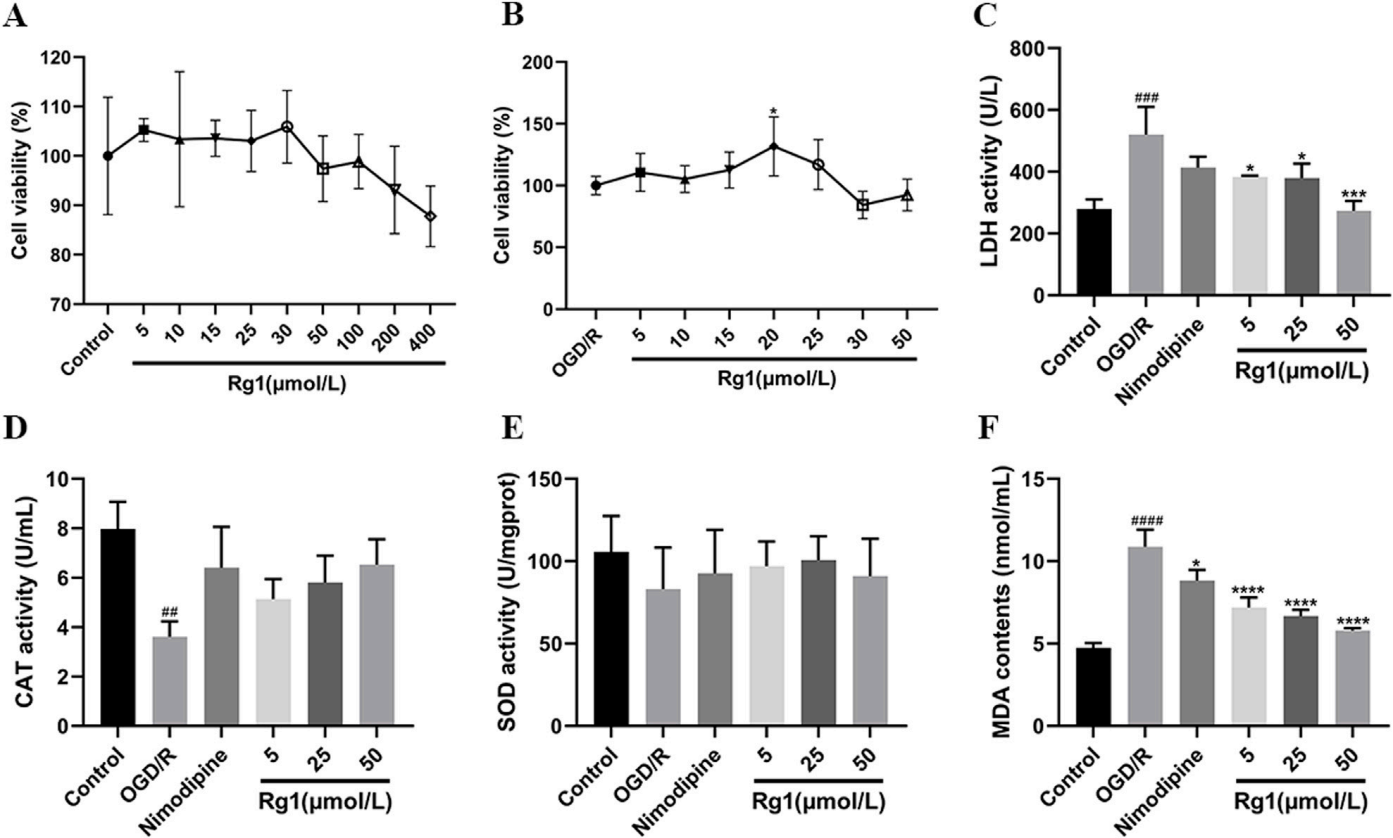
**(A)** Results of Rg1 safe concentration screening. **(B)** Results of Rg1 effective concentration screening. **(C)** Effect of Rg1 on LDH in OGD/R-injured neuronal cells (n = 3). **(D)** Effect of Rg1 on CAT in OGD/R-injured neuronal cells (n = 3). **(E)** Effect of Rg1 on SOD in OGD/R-injured neuronal cells (n = 3). **(F)** Effect of Rg1 on MDA in OGD/R-injured neuronal cells (n = 3). ## compared with control group, *P* ≤ 0.01; ### compared with control group, *P* ≤ 0.001; #### compared with control group, *P* ≤ 0.0001; * compared with OGD/R group, *P* ≤ 0.05; *** compared with OGD/R group, *P* ≤ 0.001; **** compared with OGD/R group, *P* ≤ 0.0001.

To determine the safe concentration range of Rg1, cell viability was assessed in normal HT22 cells following 24 h of incubation with varying concentrations of Rg1. The concentrations tested included 400, 200, 100, 50, 30, 25, 15, and 5 μmol/L. The results indicated that Rg1 concentrations ranging from 5 to 50 μmol/L did not significantly affect cell viability (Figure 2A). However, concentrations exceeding 100 μmol/L led to a noticeable decrease in cell viability. Based on these findings, the safe concentration range for Rg1 in subsequent experiments was established to be 5–50 μmol/L.

Following OGD/R treatment of HT22 cells, Rg1 was administered within the established safe concentration range, and its effects on cell viability and injury were assessed using the MTT assay. The results demonstrated that, compared to the OGD/R model group, Rg1 concentrations ranging from 5 to 25 μmol/L provided a protective effect, improving cell activity and reducing cell damage after OGD/R injury. Among these concentrations, 20 μmol/L of Rg1 exhibited the strongest protective effect. However, concentrations exceeding 30 μmol/L led to a significant decrease in cell viability. Based on these findings, Rg1 concentrations between 5 and 25 μmol/L were identified as the effective range for subsequent drug treatments (Figure 2B).

### 3.3 Effect of Rg1 on HT22 cells after OGD/R injury

#### 3.3.1 Effect of Rg1 on LDH activity in OGD/R-injured HT22 cells

LDH activity was significantly elevated in the OGD/R group compared to the blank control group. However, Rg1 treatment significantly reduced LDH activity relative to the OGD/R group, highlighting the inhibitory effect of Rg1 on the elevated LDH levels observed in OGD/R-damaged HT22 cells. Furthermore, the relationship between LDH activity and Rg1 concentration demonstrated a concentration-dependent trend within the safe concentration range, with higher concentrations of Rg1 exerting a more pronounced inhibitory effect on LDH activity (Figure 2C).

#### 3.3.2 Effect of Rg1 on CAT activity in HT22 cells after OGD/R injury

CAT activity was significantly reduced in the OGD/R group compared to the blank control group. However, treatment with nimodipine and high-dose Rg1 resulted in a marked increase in CAT activity relative to the OGD/R group, demonstrating the restorative effects of these interventions on CAT activity in OGD/R-injured HT22 cells. Additionally, the impact of Rg1 on CAT activity displayed a dose-dependent relationship, with higher concentrations of Rg1 leading to more pronounced restoration of CAT activity. These findings highlight the dose-dependent effect of Rg1 within its safe concentration range (Figure 2D).

#### 3.3.3 Effect of Rg1 on SOD activity in HT22 cells after OGD/R injury

SOD activity was significantly reduced in the OGD/R group compared to the blank control group. Treatment with nimodipine and varying doses of Rg1 resulted in an increase in SOD activity relative to the OGD/R group, although the differences were not statistically significant. These findings suggest that both the positive control and Rg1 may have a protective effect by mitigating the reduction in SOD levels in the supernatant of OGD/R-injured HT22 cells and partially restoring normal SOD activity *in vitro*. However, further studies are needed to comprehensively elucidate the magnitude and mechanism of this effect (Figure 2E).

#### 3.3.4 Effect of Rg1 on MDA activity after OGD/R injury in HT22 cells

MDA activity was significantly elevated in the OGD/R group compared to the blank control group, reflecting oxidative damage in HT22 cells. Treatment with nimodipine and Rg1 significantly reduced MDA levels compared to the OGD/R group, indicating their effectiveness in mitigating oxidative stress. These results suggest that both the positive control and Rg1 have potential antioxidative properties, effectively reducing MDA activity and partially restoring it to near-normal levels in OGD/R-injured HT22 cells. This highlights the potential of nimodipine and Rg1 in alleviating oxidative damage *in vitro* (Figure 2F).

#### 3.3.5 Effect of Rg1 on CKLF1 expression in HT22 cells after OGD/R injury

**FIGURE 3:**
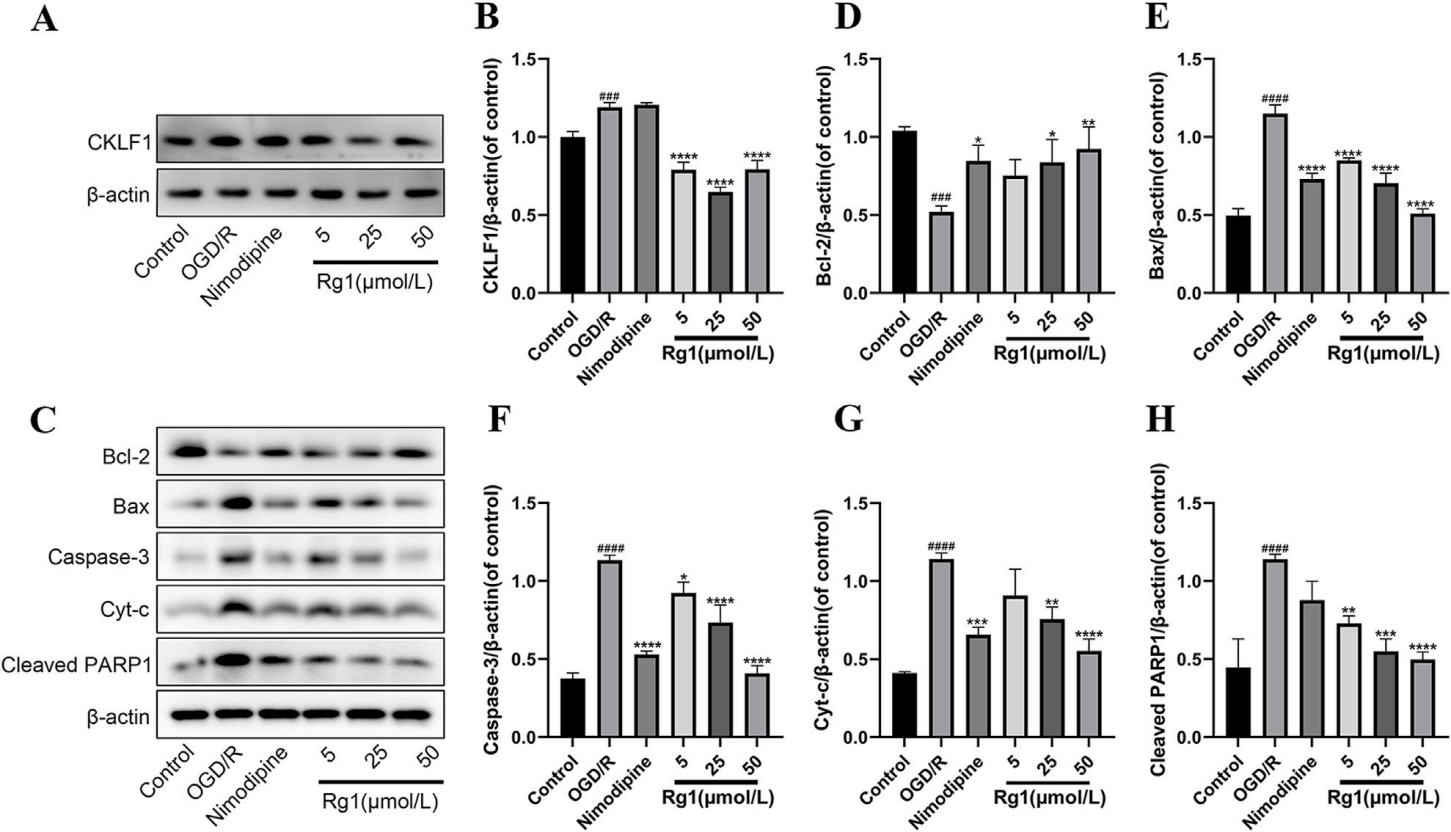
**(A)** Representative images of Western blot of CKLF1 in HT-22 cells. **(B)** Western blot statistics of CKLF1 in HT-22 cells (n = 3). **(C)** Representative images of immunoblots for Bcl-2, Bax, Caspase-3, Cyt-c and Cleaved PARP1 in HT-22 cells **(D)** Western blot statistics of Bcl-2 in HT-22 cells (n = 3). **(E)** Western blot statistics of Bax in HT-22 cells (n = 3). **(F)** Western blot statistics of Caspase-3 in HT-22 cells (n = 3). **(G)** Western blot statistics of Cyt-c in HT-22 cells (n = 3). **(H)** Western blot statistics of Cleaved PARP1 in HT-22 cells (n = 3). ### compared with control group, *P* ≤ 0.001; #### compared with control group, *P* ≤ 0.0001; * compared with OGD/R group, *P* ≤ 0.005, ** compared with OGD/R group, *P* ≤ 0.01, *** compared with OGD/R group, *P* ≤ 0.001; **** compared with OGD/R group, *P* ≤ 0.0001.

Western blot analysis demonstrated a significant increase in CKLF1 expression in the OGD/R group compared to the blank control group, indicating elevated CKLF1 levels under OGD/R conditions. Treatment with Rg1 notably reduced CKLF1 expression in HT22 cells relative to the OGD/R group, highlighting its potential to downregulate CKLF1 expression. In contrast, the positive control drug showed no significant effect on CKLF1 levels. These findings suggest that Rg1 effectively modulates CKLF1 expression in OGD/R-injured HT22 cells, potentially playing a critical role in regulating CKLF1-mediated pathways (Figures 3A, B).

#### 3.3.6 Effect of Rg1 on apoptotic protein expression after OGD/R injury in HT22 cells

Compared to the blank group, the OGD/R group exhibited a significant reduction in Bcl-2 expression, along with a marked increase in the levels of Bax, Caspase-3, Cyt-c, and Cleaved PARP1. However, treatment with both nimodipine and Rg1 led to a significant upregulation of Bcl-2 and a marked downregulation of Bax, Caspase-3, Cyt-c, and Cleaved PARP1 relative to the OGD/R group (Figures 3C–H). These findings suggest that nimodipine and Rg1 confer protective effects on OGD/R-injured HT22 cells by modulating the expression of key apoptotic markers, including Bcl-2, Bax, Caspase-3, Cyt-c, and Cleaved PARP1.

#### 3.3.7 Effect of Rg1 on CKLF1 fluorescence expression in HT22 cells after OGD/R injury

**FIGURE 4:**
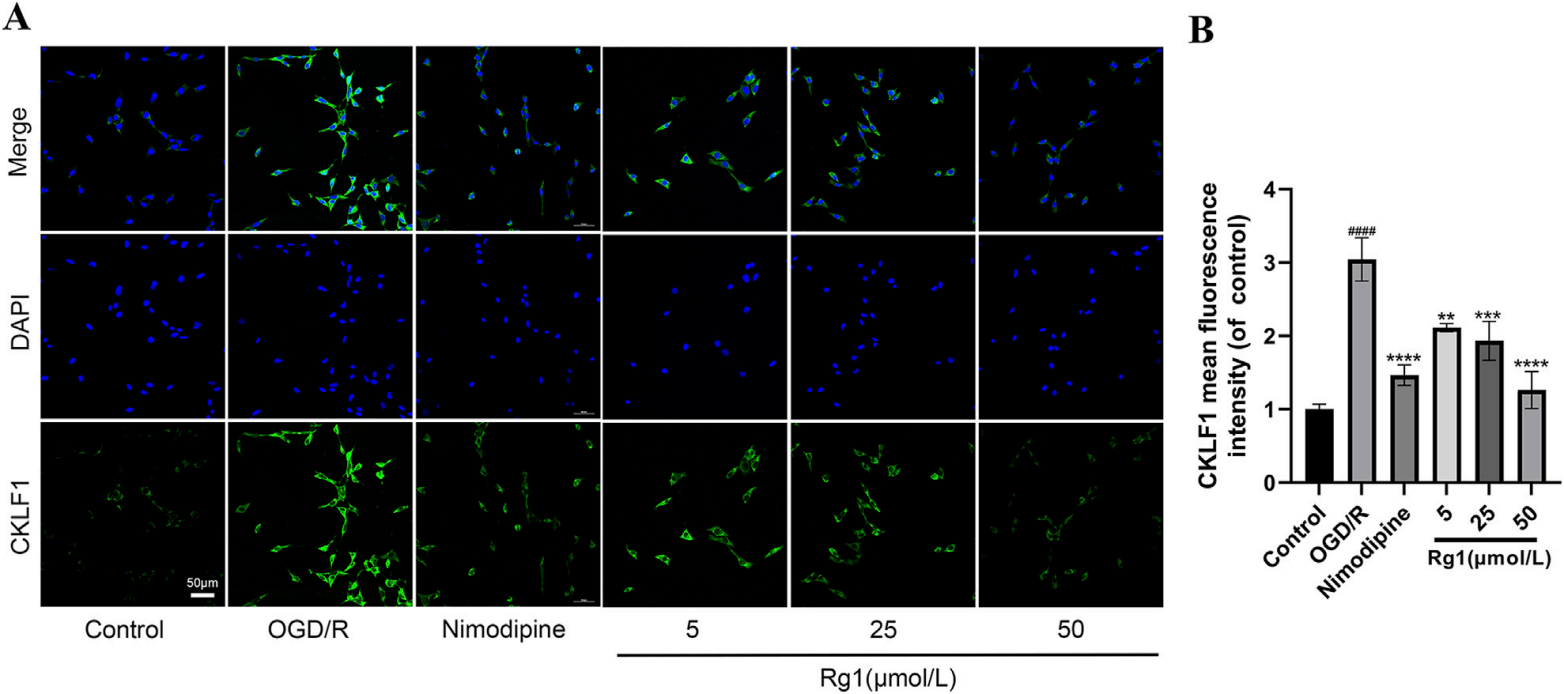
**(A)** Representative images of IF staining of CKLF1 in HT22 cells (×400 magnification, scale bar = 50 μm). **(B)** Statistical results of IF staining of CKLF1 in HT22 cells (n = 3). #### compared with control group, *P* ≤ 0.0001; ** compared with OGD/R group, *P* ≤ 0.01; *** compared with OGD/R group, *P* ≤ 0.001; **** compared with OGD/R group, *P* ≤ 0.0001.

IF staining revealed a significant increase in the fluorescent expression of CKLF1 in OGD/R-injured HT22 cells. However, treatment with Rg1 and nimodipine resulted in a marked reduction in CKLF1 expression, suggesting that both Rg1 and nimodipine exert protective effects on HT22 cells by inhibiting the upregulation of CKLF1 (Figures 4A, B).

#### 3.3.8 Effect of Rg1 on apoptosis after OGD/R injury in HT22 cells

**FIGURE 5:**
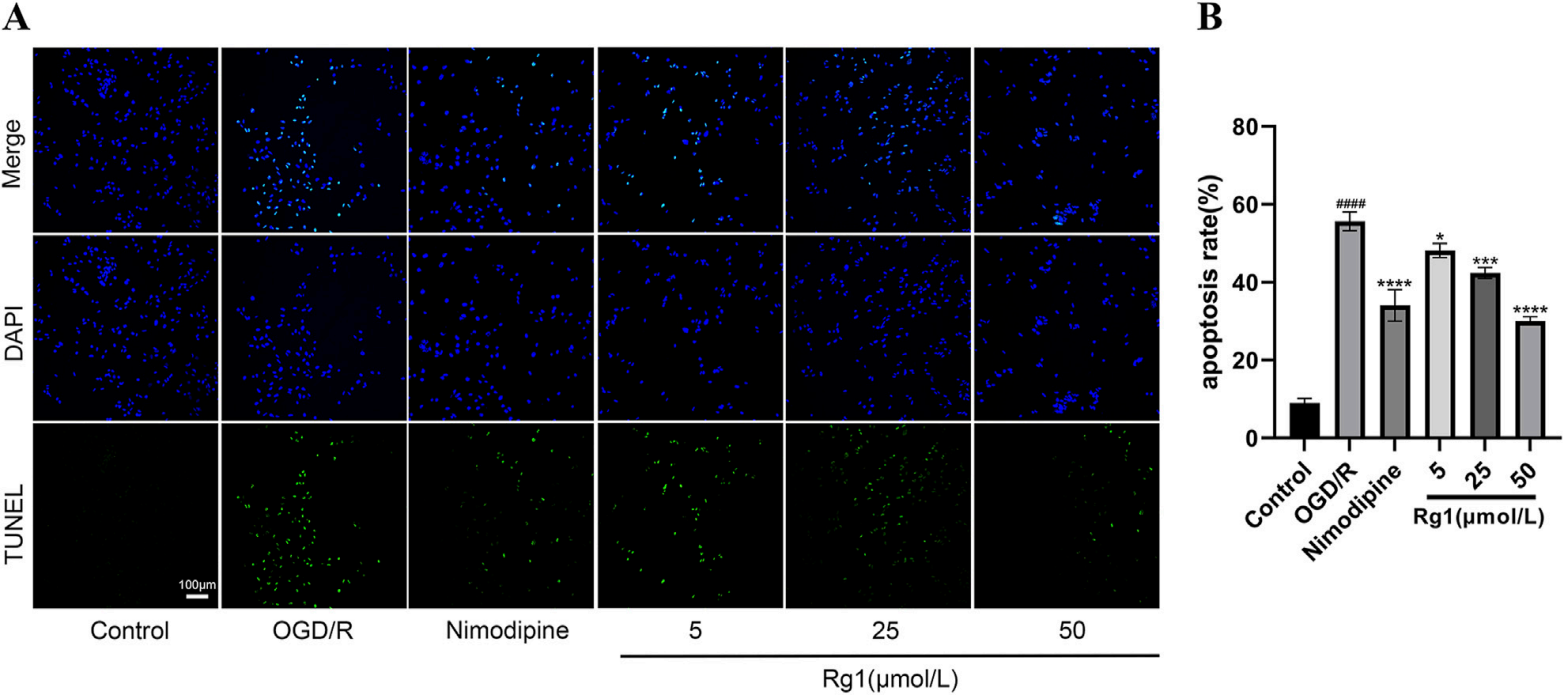
**(A)** Changes of the apoptotic rates in HT22 cells by OGD/R with different treatments examined by TUNEL staining (×200 magnification, scale bar = 100 μm), DAPI: bule; TUNEL: green; **(B)** graphical representation of apoptosis rate by TUNEL staining (n = 3). #### compared with control group, *P* ≤ 0.0001; * compared with OGD/R group, *P* ≤ 0.05; *** compared with OGD/R group, *P* ≤ 0.001; **** compared with OGD/R group, *P* ≤ 0.0001.

TUNEL staining revealed significant apoptosis in HT22 cells following OGD/R treatment. However, both Rg1 and nimodipine effectively inhibited apoptosis in OGD/R-injured HT22 cells, with 50 μmol/L Rg1 exhibiting the most pronounced inhibitory effect on cell apoptosis (Figures 5A, B).

### 3.4 Effect of Rg1 on inflammatory factors in BV-2 cells after OGD/R injury

#### 3.4.1 Effect of Rg1 on NO of microglia in co-culture system

**FIGURE 6:**
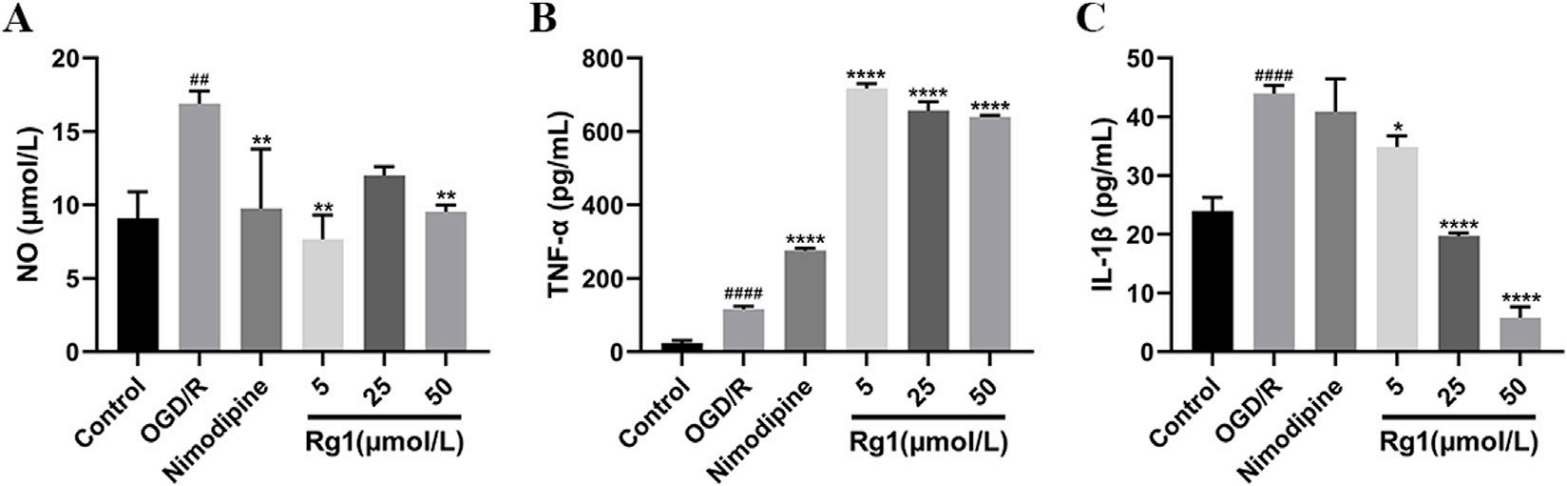
**(A)** Effect of Rg1 on NO in BV-2 cells in co-culture (n = 3). **(B)** Effect of TNF-α on NO in BV-2 cells in co-culture (n = 3). **(C)** Effect of Rg1 on IL-1β in BV-2 cells in co-culture (n = 3). ## compared with control group, *P* ≤ 0.01; #### compared with control group, *P* ≤ 0.0001; * compared with OGD/R group, *P* ≤ 0.05; ** compared with OGD/R group, *P* ≤ 0.01; **** compared with OGD/R group, *P* ≤ 0.0001.

The NO content was significantly elevated in the OGD/R group compared to the blank group. However, treatment with Rg1 resulted in a marked reduction in NO levels, with the low-dose Rg1 group exhibiting the most pronounced inhibitory effect. These findings suggest that Rg1 effectively attenuates the excessive production of NO in OGD/R-injured HT22 cells, indicating its potential to regulate NO-mediated cellular responses (Figure 6A).

#### 3.4.2 Effect of Rg1 on TNF-α of microglia in co-culture system

The level of TNF-α was significantly elevated in the OGD/R group compared to the blank group, reflecting an increased inflammatory response. Notably, treatment with nimodipine further exacerbated TNF-α levels relative to the OGD/R group. Similarly, Rg1 administration across various concentrations did not reduce TNF-α levels; instead, it significantly increased TNF-α production. Among the Rg1-treated groups, the low-dose Rg1 group exhibited the highest secretion of inflammatory factors. These findings suggest that Rg1 may exert a complex and potentially pro-inflammatory influence on TNF-α regulation in OGD/R-injured HT22 cells, underscoring the need for further investigation to elucidate its underlying mechanisms (Figure 6B).

#### 3.4.3 Effect of Rg1 on IL-1β of microglia in co-culture system

The level of IL-1β was significantly elevated in the OGD/R group compared to the blank group, indicating a heightened inflammatory response. Treatment with nimodipine resulted in only a slight reduction in IL-1β levels relative to the OGD/R group. In contrast, Rg1 administration across varying doses significantly reduced IL-1β levels in a dose-dependent manner, with higher doses of Rg1 demonstrating greater suppression of IL-1β production. These findings suggest that Rg1 possesses notable anti-inflammatory properties by attenuating IL-1β production in OGD/R-injured HT22 cells. Further research is warranted to investigate the mechanisms underlying Rg1-mediated regulation of IL-1β (Figure 6C).

## 4 Discussion

*In vitro* studies on cerebral ischemia-reperfusion disease widely recognize the OGD model as an ideal experimental platform for investigating this condition at the cellular level. To develop a well-defined and relevant hypoxia-reperfusion model suited to laboratory conditions, we systematically optimized key parameters, including an OGD/reperfusion duration of 2.5 h/2.5 h, a cell seeding density of 1 × 10^5^ cells/mL, a concentration of 25 mmol/L for the anoxic agent Na_2_S_2_O_4_, and a 24-h incubation period. Nimodipine was utilized as the positive control drug for comparative analysis. Using high-throughput screening technology and relevant analytical formulae, we obtained robust data validating effectiveness of the model. The reliability of the OGD/R model was further confirmed through assessments of cell survival rates, LDH levels, and other relevant biomarkers. These findings demonstrate the model’s suitability for various applications, including primary drug screening, mechanistic exploration, and safety evaluations in the context of neuroprotective drug development. In order to establish a robust cellular model for the subsequent study of the mechanism of action of Rg1 on neurological ischemia-reperfusion injury, one-way experiments were performed to identify and prioritise key modelling factors. The results indicated significant differences among the screened parameters, with the concentration of the chemical hypoxic agent emerging as the most influential factor. These results provide a solid basis for selecting optimal modelling parameters and advancing research into the therapeutic potential of Rg1.

In this experimental study, we first investigated the effects of different concentrations of Na_2_S_2_O_4_ on cell survival rates. We observed that concentrations below 10 mmol/L had minimal impact on cell viability. Based on these findings, we adjusted the hypoxic agent concentration for further screening. As shown in Figure 1, a concentration of 25 mmol/L resulted in approximately 50% cell survival, indicating an effective modeling effect. To optimize experimental conditions, we considered both the cell density and hypoxic agent concentration as individual factors. Given that higher seeding densities resulted in cell clumping and lower densities caused potential cell damage, we systematically examined these variables. By optimizing these four key factors, we successfully established a validated OGD/R model using the Z-factor method for high-throughput screening. Nimodipine was included as a positive control, and the Z-factor value exceeding 0.5 further supported the accuracy and reliability of the model. These results not only validate our experimental approach but also lay a solid foundation for future studies, reinforcing the credibility of our research methodology.

Oxidative stress, an imbalance between free radicals and the antioxidant system leading to the overproduction of oxygen radicals, is an early event in hypoxic reoxygenation conditions and plays a crucial role in the cellular response to hypoxia. Hypoxic conditions can trigger the production of oxygen free radicals, which are closely associated with cellular structure and morphology. The excessive accumulation of free radicals can lead to increased cellular mobility and permeability. Pharmacological intervention targeting oxidative stress has been recognized as a key approach in the treatment of cerebral ischemia-reperfusion injury (Briyal et al., 2023; Yoshikawa and You, 2024). During cerebral ischemia, a large number of reactive oxygen species are generated, overwhelming the cellular antioxidant defense mechanisms such as SOD, CAT, and GSH-Px (Sun et al., 2023). This imbalance between free radical production and antioxidant capacity leads to oxidative stress, which triggers oxidative modification of biomolecules (including lipids, proteins and nucleic acids), leading to cellular dysfunction and damage (Li J. et al., 2023). The major oxidative stress molecules, including superoxide anions, hydroxyl radicals, hydrogen peroxide, nitric oxide, and peroxynitrite, can all contribute to cellular structural damage in the presence of oxidative stress. Upon the onset of cerebral ischemia, mitochondria generate a substantial amount of oxygen radicals. As mitochondrial function deteriorates, the production of oxygen radicals gradually decreases. Concurrently, ATP metabolites activate xanthine oxidase, leading to the generation of additional oxygen radicals. Furthermore, calcium ions activate reduced nicotinamide adenine dinucleotide phosphate oxidase, which further contributes to oxygen radical production.

The results presented in Figures 2C, D, F demonstrate significant alterations in the activity levels of LDH, MDA, and CAT in the supernatants of HT22 cells subjected to OGD/R injury. In contrast, no significant changes were observed in the activity of SOD. These findings provide evidence of cellular damage and oxidative stress induced by OGD/R. Furthermore, Figures 2C, D, F show that Rg1 treatment effectively inhibits the increases in LDH and MDA activity while restoring the decrease in CAT activity caused by OGD/R. These results suggest that Rg1 exerts a comprehensive protective effect in the OGD/R model, primarily through its antioxidant properties, leading to the inhibition of apoptosis. Additionally, Figures 3A, B reveal a significant upregulation in the expression of CKLF1 protein following hypoxia-reoxygenation. This increase, coupled with elevated levels of LDH and MDA and decreased SOD and CAT activity, suggests that CKLF1 is involved in the oxidative stress response triggered by OGD/R. Notably, Rg1 administration resulted in a significant decrease in CKLF1 expression compared with the blank and model groups, highlighting the inhibitory effect of Rg1 on CKLF1 expression in HT22 cells after hypoxia and reoxygenation. These findings suggest that CKLF1 may serve as a potential target in the cascade of injuries associated with hypoxic reoxygenation, and Rg1 effectively reduces its expression. Consequently, Rg1 emerges as a promising CKLF1 inhibitor, exerting anti-oxidative stress effects to protect neuronal cells following injury. To further investigate the potential involvement of CKLF1 in Rg1-mediated apoptosis, we examined the expression levels of key apoptotic proteins. The results indicated significant changes in the expression of apoptosis-related proteins, including Bcl-2, Bax, Caspase-3, Cyt-c, and Cleaved PARP1 in the OGD/R group compared to the blank group. Specifically, Rg1 treatment increased Bcl-2 expression and decreased the expression of pro-apoptotic proteins such as Bax, Caspase-3, Cyt-c, and Cleaved PARP1 when compared to the OGD/R group (Figures 3C–H). These findings suggest that HT22 cells subjected to OGD/R injury undergo an oxidative stress response, which leads to increased apoptosis. Bcl-2 and Bax are key regulators of apoptosis that modulate mitochondrial membrane permeability. Bcl-2 is an anti-apoptotic protein expressed predominantly in mitochondria that maintains calcium homeostasis, inhibits the opening of the mitochondrial permeability transition pore, and prevents the release of pro-apoptotic factors. In contrast, Bax, a pro-apoptotic protein, is essential for the formation of the mitochondrial membrane pore during apoptosis and forms a heterodimer with Bcl-2 to promote apoptosis (Chen et al., 2015; Xu and Ye, 2022). The observed increase in Bcl-2 levels and decrease in Bax levels suggest enhanced resistance to apoptosis, with the Bcl-2/Bax ratio serving as a key indicator of apoptotic regulation. Activation of the Bcl-2/Bax complex triggers the release of apoptotic protease-activating factors from the mitochondrial intermembrane space, activating Caspase-3 and initiating the apoptotic cascade. Caspase-3 is a central component of the apoptotic signaling pathway, playing a pivotal role in the execution of apoptosis. Additionally, Cyt-c, an electron transport protein in the mitochondrial respiratory chain, is released into the cytoplasm following mitochondrial membrane permeabilization. In the cytoplasm, Cyt-c binds to apoptotic protease-activating factor-1, leading to caspase activation and the initiation of the intrinsic apoptotic pathway (Pessoa, 2022). Furthermore, during the execution phase of apoptosis, cleaved poly (ADP-ribose) polymerase (Cleaved PARP1) impairs DNA repair mechanisms, promoting cell death (Diamantopoulos et al., 2014).

In this experiment, a neuron-microglia co-culture system was established to more accurately simulate the pathological processes associated with cerebral ischemia-reperfusion injury. This system not only mimics the smallest functional unit of the nervous system but also represents aspects of the BBB, allowing for a more comprehensive model to investigate the underlying mechanisms of this pathology. Co-culture systems enable the observation of cellular communication, facilitate the exploration of drug mechanisms, aid in the identification of potential therapeutic targets, and support the differentiation of cells into specific cell types (Liu et al., 2022). The CNS primarily consists of neurons and glial cells, and their interactions are critical in response to injury. Neurons release various cytokines in response to damage, which can influence microglial activation. Microglia, the resident immune cells of the CNS, can polarize into two distinct phenotypes: the pro-inflammatory M1 phenotype and the anti-inflammatory M2 phenotype (Kwon and Koh, 2020). Following cerebral ischemia, immune cells, including microglia, undergo activation, transitioning from the M2 to the M1 phenotype, which leads to the production of pro-inflammatory cytokines such as IL-1β, IL-6, TNF-α, and chemokines. These factors are key markers for diagnosing and prognosticating ischemic damage but can exacerbate neuronal injury, impairing neuronal proliferation and differentiation (Zeng et al., 2018; Jayaraj et al., 2019). The experimental results demonstrated that HT22 cells, when subjected to ischemic injury, activated BV-2 microglial cells and promoted the production of inflammatory cytokines. However, when Rg1 was introduced into the co-culture system, the detrimental effects of OGD/R on HT22 cells were significantly mitigated. As shown in Figures 6A,C, Rg1 treatment primarily reduced the expression levels of nitric oxide (NO) and IL-1β. Interestingly, Figure 6B revealed an increase in the expression of TNF-α upon Rg1 intervention. TNF-α, a critical inflammatory mediator, can induce cell death in tumor cells but generally has less toxicity to normal cells. Elevated TNF-α levels are indicative of a severe inflammatory response.

In summary, the use of a neuron-microglia co-culture system in this study provided valuable insights into the complex inflammatory interactions involved in cerebral ischemia-reperfusion injury. Rg1 intervention demonstrated its ability to reduce HT22 cell damage and modulate the expression of key inflammatory factors. These findings contribute to our understanding of the potential of Rg1 as a therapeutic agent to mitigate the inflammatory response and protect neuronal cells under ischemic conditions.

The experimental findings demonstrate that Rg1 effectively mitigates the inflammatory response of microglia in the neuron-microglia co-culture system, thereby preserving the stability of the neuronal environment and exerting a protective effect on neurons. However, the precise mechanisms underlying this protective effect remain to be fully elucidated and warrant further investigation. Additional studies are necessary to identify and clarify the specific molecular pathways and signaling cascades through which Rg1 modulates the inflammatory response and promotes neuroprotection. A more comprehensive understanding of the mechanistic basis of Rg1 action is essential for the development of targeted therapeutic strategies aimed at managing cerebral ischaemia-reperfusion injury.

## 5 Conclusion

**FIGURE 7:**
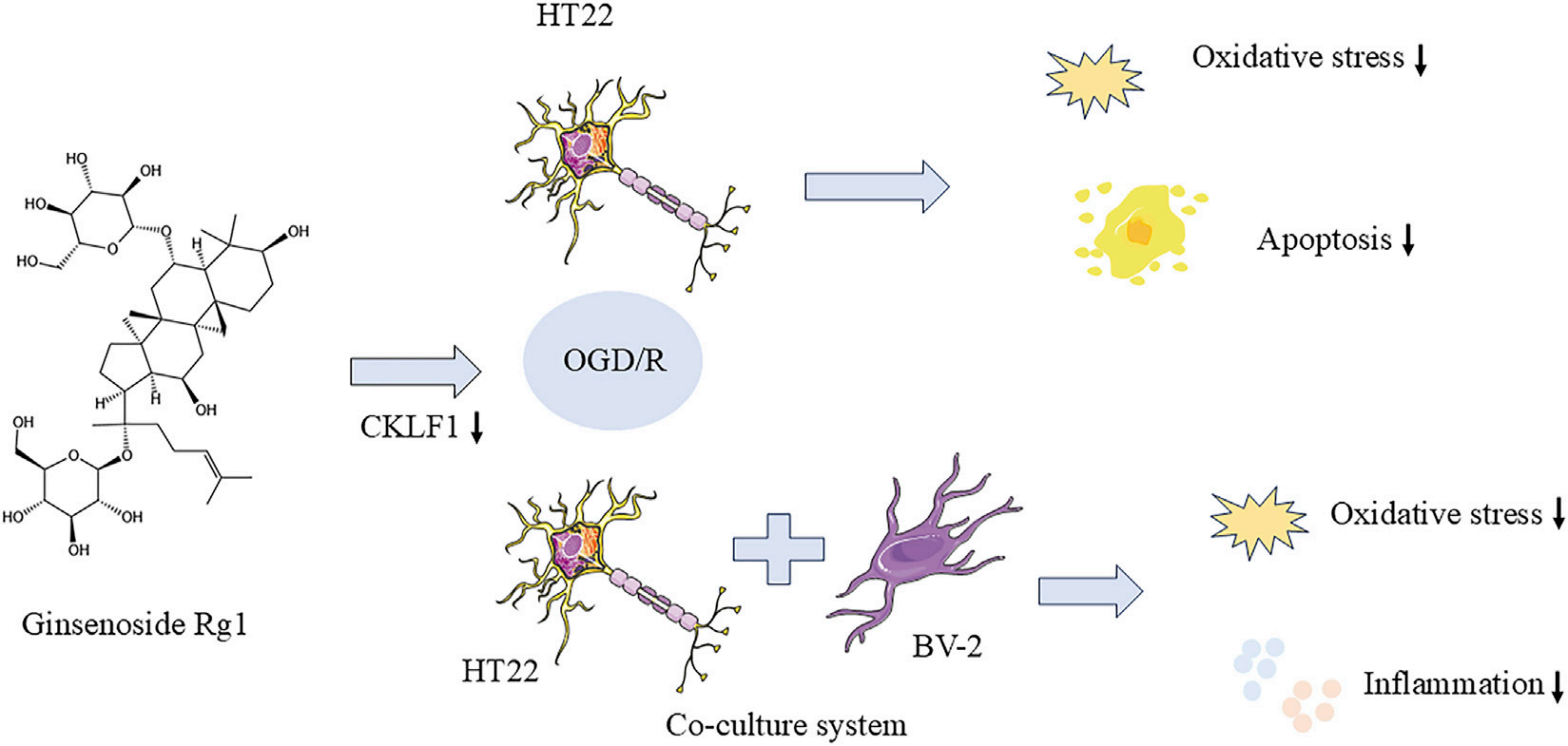
Rg1 inhibited oxidative stress and apoptosis in HT22 cells after OGD/R injury.

Rg1 exhibits inhibitory effects on oxidative stress and apoptosis in HT22 cells following OGD/R injury. It appears that CKLF1 may play a role in the oxidative stress process during hypoxia-reoxygenation, with Rg1 potentially exerting its neuroprotective effect by suppressing CKLF1 expression in HT22 cells after OGD/R injury. This suppression of CKLF1 expression contributes to the attenuation of oxidative stress and the inhibition of apoptosis. Additionally, the neuron-microglia co-culture system demonstrated that Rg1 significantly reduces the production of inflammatory factors in BV-2 cells, further supporting its neuroprotective effects following OGD/R injury (Figure 7). These findings suggest that Rg1 holds promise as a potential therapeutic agent for mitigating neuronal damage and promoting neuroprotection in the context of cerebral ischemia-reperfusion injury. However, further studies are needed to fully elucidate the exact mechanism of Rg1 action and to explore its clinical implications for the treatment of neurological disorders.

## Data Availability

The raw data supporting the conclusions of this article will be made available by the authors, without undue reservation.
